# Hepatitis C Virus Infection and Lymphoma

**DOI:** 10.4084/MJHID.2010.004

**Published:** 2010-03-31

**Authors:** Emmanuel Bachy, Caroline Besson, Felipe Suarez, Olivier Hermine

**Affiliations:** 1 Service d’hématologie adulte, Hôpital Necker-Enfants Malades, Assistance Publique-Hôpitaux de Paris, Université René Descartes Paris V, CNRS UMR 8147 Paris, France; 2 Service d’hématologie Biologique, Hôpital du Kremlin Bicêtre, Université Paris Sud, France

## Abstract

Apart from its well known role as an etiological agent for non-A and non-B viral hepatitis, there is growing evidence that hepatitis C virus is associated to B-cell non-Hodgkin lymphoma. The association between HCV and lymphoproliferative disorders has been recently postulated based on epidemiological data, biological studies and clinical observations. Although various subtypes of lymphomas appear to be associated to HCV, diffuse large B-cell lymphoma, small lymphocytic lymphoma/chronic lymphocytic leukemia and marginal zone lymphoma appeared to be particularly represented among HCV-positive patients. The causative role of HCV in those disorders has been further supported by the response to antiviral therapy. Despite a better understanding of pathophysiological processes at stake leading from HCV infection to overt lymphoma, many issues still need to be further elucidated. Although HCV has been demonstrated to directly infect peripheral blood mononuclear cells both *in vitro* and, in some cases, *in vivo*, a strong body of evidence rather supports the hypothesis of an indirect transformation mechanism by which sustained antigenic stimulation leads from oligoclonal to monoclonal expansion and sometimes to lymphoma, probably through secondary oncogenic events. Here, we review epidemiological and biological studies, as well as clinical data on antiviral therapy, linking HCV-infection to B-cell non-Hodgkin lymphoma.

## Introduction:

Hepatitis C virus (HCV) is a small (∼9600 nucleotide) encapsulated positive strand RNA member of the *Flaviviridae* family. The virus lacks a reverse-transcriptase and its genome encodes a single open reading frame for a large polyprotein, which is subsequently cleaved to structural and non-structural (enzymatic) component viral proteins. For more than a decade, evidence from either epidemiological studies, therapeutic approaches or biological data have emerged giving strong support to an etiological role of HCV in non-Hogkin lymphoma (NHL) development 1,2.

Thus, many large case-control studies have reported a clear association between HCV infection and NHL development. Furthermore, HCV-associated NHL response to interferon (IFN) and ribavirin therapy in case of viral load decrease, previously described in several studies, also gave strong support for an etiopathological role of HCV in this kind of lymphoproliferative disorder.

Although HCV is the major etiologic agent of non-A and non-B chronic hepatitis, it can present with a broad spectrum of extrahepatic manifestations. Among them, immune-related disorders have been described such as type II mixed-cryoglobulinemia (MC), characterized by a monoclonal IgM with rheumatoid factor (RF) activity (*i.e.* an IgM with anti-IgG activity) with or without an overt cryoglobulinemia-associated vasculitis. About 8 to 10% of patients with MC ultimately develop a frank lymphoproliferative disorder and recent data demonstrated a 35 times increased risk of lymphoma for cryoglobulinemia carriers. Among pathogenetic hypotheses, HCV lymphotropism has been studied but no direct transformation leading to an overt lymphoproliferative disorder has been clearly demonstrated. Conversely, strong evidence for an indirect role for HCV in inducing lymphoproliferative disorders has been given by recent findings. In this regard, association between HCV-infection, mixed cryoglobulinemia (MC) and NHL lent support to a multistep model in which HCV would induce a protracted stimulation of antigen-specific B-cell clones, leading to MC and in a subset of patients to overt lymphomas.

Thus, cumulative evidence for a pathological link between HCV infection and lymphoma has emerged and will be reviewed in this paper.

## HCV-associated lymphoma: evidence from epidemiological studies

Several epidemiological studies have been performed to establish a link between HCV infection and NHL. Early studies based on relatively small number of patients provided conflicting results and suggested a significant increased risk of B-NHL in HCV-infected patients only in high prevalence areas.^3^,^4^ Discrepancies concerning HCV prevalence in lymphoma patients demonstrated in case-control studies from North America and North European countries have thus been pointed out. In those studies, the prevalence of HCV infection among patients with NHL did not significantly differ from controls. This might be explained, at least in part, by a large difference in HCV prevalence itself (which is lower in those countries), or by yet unknown environmental and genetic factors.^5^,^6^ Two large US studies involving the NCI-SEER registry and the US Veterans Affairs health system respectively, as well as an analysis by the European multicentre EPILYMPH consortium, have documented a positive, modestly increased risk of NHL in patients with HCV as compared to HCV-negative controls (relative risk (RR)=2–3).^7^,^8^ These latter studies were additionally supported by a concurrent meta-analysis review of 15 case-control and 3 prospective studies in this field, showing a “pooled” RR of 2–2.5 depending on study design.^9^ Eventually, another recently published meta-analysis included 7 member studies from the International Lymphoma Epidemiology Consortium (InterLymph) based in Europe, North America, and Australia.^10^ HCV infection was detected in 3.60% of NHL cases and in 2.70% of controls (odds ratio [OR], 1.78; 95% confidence interval [CI], 1.40–2.25). It thus appears that there is a greater propensity to develop NHL in the setting of HCV infection, and that the risk is most dramatically evident in populations with high HCV prevalence. Besides, geographic variability worldwide may indicate additional important environmental factors influencing the strength of this relationship. In addition, providing further evidence, a report from Japan demonstrated that 2,5% of patients infected by the virus were at risk of developing NHL within a 15-year period of follow-up. Interestingly, during the same period following antiviral therapy, none of the patients who cleared the virus developed NHL whereas those who remained PCR positive had the same risk as not treated patients.^11^

Not all lymphoma subtypes are equally represented among those lymphoproliferative disorders. Hence, splenic (with or without villous lymphocytes) or non splenic marginal zone lymphomas (MZL), diffuse large B-cell lymphomas (DLBCL), but not follicular lymphoma (FL) or T-cell lymphomas have been associated with HCV infection. Hence, the recent meta-analysis from the InterLymph Consortium demonstrated that, in subtype-specific analyses, HCV prevalence was associated with MZL (OR, 2.47; 95% CI, 1.44–4.23), DLBCL (OR, 2.24; 95% CI, 1.68–2.99), and lymphoplasmacytic lymphoma (OR, 2.57; 95% CI, 1.14–5.79) whereas risk estimates were not increased for FL (OR, 1.02; 95% CI, 0.65–1.60).^10^ Thus, the most common lymphomas found in patients with HCV are low grade MZL particularly those of splenic origin and high-grade DLBCL with extranodal localizations.^11^–^13^ These lymphoproliferations exhibit peculiar features: i. they usually occur following a long period of infection (more than 15 years), ii. are not associated with a specific virus genotype, iii. often involve extranodal sites particularly liver, spleen and salivary glands.

However, despite these epidemiological and clinical evidences, the role of HCV in lymphomagenesis has remained elusive, and only recently have pathophysiological models started to emerge.

## HCV-associated lymphoma: evidence from antiviral therapy efficacy on lymphoma

**Figure 1.**
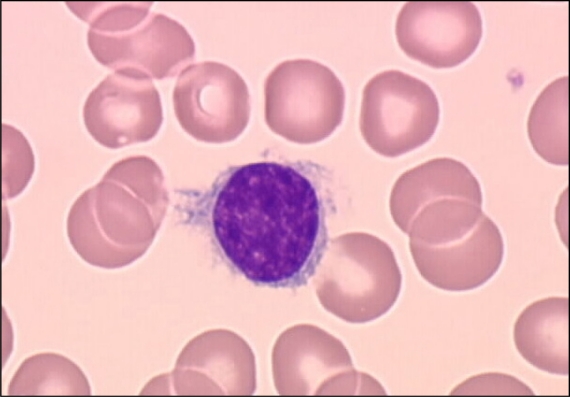
**Villous lymphocyte.** Blood smear showing a villous lymphocyte with conspicuous cytoplasmic villous protrusions from a patient with splenic lymphoma with villous lymphocytes (SLVL). May-Grünwald-Giemsa stain, X1000.

If HCV infection has been inferred to be a factor in the development of NHL on the basis of case-control epidemiological studies as previously discussed, strong line of evidence also arose from response of so called HCV-associated lymphoma to antiviral therapy. Hence, in 2002, we reported the outcome of 9 patients who had splenic MZL with villous lymphocytes and HCV infection treated with interferon alfa-2b (IFN) alone or in combination with ribavirin.^14^ Splenic lymphoma with villous lymphocytes (SLVL) is a clonal chronic B-cell lymphoproliferative disorder characterized by splenomegaly and peripheral blood malignant circulating B lymphocytes with villous projections (**Figure 1**). Histologically, the lymphoid marginal zone surrounding the follicular areas is expanded by neoplastic cells that have cytological and phenotypical features of marginal zone B cells.^15^ As a consequence, clonal expansion of villous lymphocytes is supposed to originate from the marginal zone of the spleen although definitive conclusion has not been clearly demonstrated to date. From a clinical point of view, the disease displays an indolent evolution with splenomegaly increasing over years and with a gradual progression of circulative malignant B cells. Of the 9 IFN-treated patients, 7 achieved a complete hematological remission, defined by the absence of abnormal lymphocytosis and the resolution of the splenomegaly, after HCV RNA load became undetectable. The remaining 2 patients experienced a partial or a complete response after addition of ribavirin and the loss of detectable HCV RNA. Conversely, none of 6 similarly treated HCV-negative SLVL patients responded to therapy thereby suggesting that the observed response rate was not due to the effect of interferon itself. Those results gave support to the hypothesis that HCV might trigger, to some extent, clonal expansion and oncogenic events at least in a subgroup of indolent lymphomas patients. We also reported in 2005 on a series of 18 patients with HCV-associated SLVL.^16^ All patients had MC, the majority of whom having symptomatic MC (72%), a much higher proportion than HCV-infected patients without SLVL. Apart from this finding, HCV-positive SLVL did not differ from HCV-negative SLVL patients. All patients were treated with alpha-IFN with (10 patients) or without (8 patients) ribavirin. Four patients had received prior therapy for SLVL including splenectomy or chemotherapy. Six patients received associated therapy for symptomatic MC (steroids, cyclophosphamide or plasmapheresis). Complete hematological remission was observed in 78% of the patients, most of them having concomitant complete virological responses (*i.e.* disappearance of HCV RNA). Two patients with major virological responses (more than 2 log reduction in HCV RNA) also achieved complete hematological remission, whereas the 2 patients with minor virological responses (less than 2 log reduction in HCV RNA) only achieved partial hematological responses (*i.e.* reduced albeit persistent circulating villous lymphocytes and splenomegaly). Moreover, in one patient with a virological relapse, villous lymphocytosis reappeared, but re-initiation of antiviral therapy was associated with a second complete hematological remission following HCV RNA reduction. The mean time to treatment responses was approximately 4 months for both virological and hematological responses. Mean duration of antiviral treatment was 17 months. Responses were sustained, as the mean duration of hematological response was 62 months. Clinical manifestations of MC subsided in all patients after antiviral treatment. Interestingly, viral genotype did not seem to correlate with the response as 4 out of 7 patients presenting with HCV genotype 1, usually associated with poor responses, achieved a complete hematologic response. Of note, even for patients who exhibited a complete hematological remission, B-cell clone could still be detected in peripheral blood but clinical relapses did not occur if viremia remained negative.

Overall, these observations strongly supported a causal relationship between HCV replication and lymphomagenesis in SLVL. Another report confirmed those results showing that among 8 patients with MZL of MALT or splenic (with or without villous lymphocytes) subtypes, alpha-IFN and ribavirin yielded a 60% response rate, which was correlated to virologic response in most cases.^17^ A more recently published study extended these results by showing efficacy of antiviral therapy in other histological subtypes of indolent NHL associated with HCV infection, including follicular lymphoma and splenic MZL.^18^

Altogether, response to antiviral therapy in HCV-related NHL in case of viral load decrease gave further insights into the pathogenesis of those disorders and strengthened the association presumed from epidemiological studies.

## HCV-associated lymphoma: evidence from biological studies

**Figure 2.**
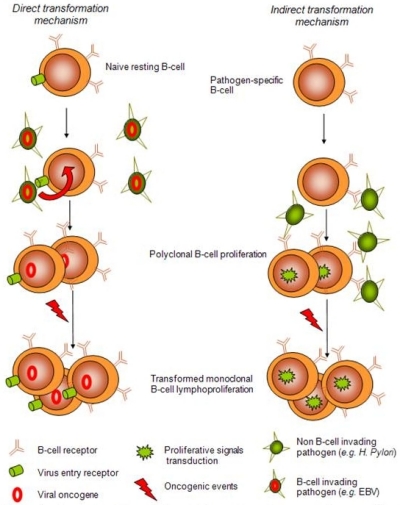
**General models of lymphoid transformation by pathogens. Direct transformation model.** Infectious agents such as Epstein-Barr virus directly target resting B-cell and establish latent infection. Transcription of viral latent genes with oncogenic potential leads to immortalization of infected B-cell and proliferation, normally kept in check by the immune system of the host. Under certain circumstances (as immune deficiency) or after additional oncogenic mutations, fully transformed EBV-infected B-cell might lead to malignant lymphoma. **Indirect transformation model.** Persisting pathogens such as *Helicobacter Pylori* in chronic infection stimulate antigen-specific B-cell either directly or indirectly through T-cell help. Clonal expansion may develop in responding lymphocytes sometimes ultimately leading to frank lymphoproliferation.

Although epidemiological studies and clinical data link NHL with HCV, underlying biological processes ultimately leading from infection to lymphoma are still poorly understood. Basically, relationships between infectious agents and lymphoproliferative disorders such as *Helicobacter pylori* and gastric MALT-lymphoma, Epstein-Barr virus (EBV) and lymphoproliferative disorders (LPD), human herpesvirus 8 (HHV8) and primary effusion lymphoma (PEL) or human T-lymphotropic virus 1 (HTLV-1) and adult T-cell leukemia/lymphoma have been extensively studied for the two last decades but no clear pathological model has emerged so far. Nevertheless, antigen-driven lymphoproliferation might be though of as being split into 2 distinct mechanisms. On one hand, direct lymphocyte transformation by a given agent such as lymphotropic transforming viruses (EBV, HHV8, or HTLV1) expressing viral oncogenes has been clearly demonstrated.^19^–^21^ On the other hand, a more recently described model for an indirect transformation mechanism of lymphocytes ultimately leading to clonal expansion has emerged, among which *Helicobacter Pylori*-associated gastric MALT lymphoma might be the best characterized (**Figure 2**).^22^

As a matter of fact, growing evidence show that, when exposed to chronic antigenic stimulation, B cells accumulate genetic lesions through inherent genomic instability during activation-induced deaminase (AID)-mediated variable-determining-joining V(D)J class switch recombination (CSR) and/or somatic hypermutation (SHM).^23^ Both of these reactions have been shown to produce double-strand DNA break intermediates that might be aberrantly resolved as chromosomal translocations.^24^,^25^

Several oncogenes have been described as targets for somatic hypermutation or translocations with immunoglobulin heavy-chains regions (IgH). In most cases, these anomalies alarm the DNA damage response system that either allows for DNA repair or eliminates the aberrant B-cell clones.^19^ Occasionally, those repair systems fail and B-cell malignancies may arise.

**Figure 3.**
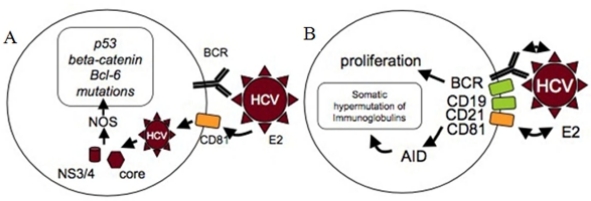
**Hypothetical model of B-cell transformation by HCV. 3A. Direct transformation model.** Induction of cellular NO-synthase and subsequent mutations of p53, beta-catenin and Bcl-6 by HCV associated core and NS3/4 proteins may participate in B-cell transformation. According to this hypothesis, HCV would directly infect B cells, possibly through CD81-E2 interaction. **3B. Indirect transformation model.** Interaction of E2 and CD81 on the cell surface induces expression of activation-induced deaminase (AID) and somatic hypermutations of immunoglobulin genes and potential proto-oncogenes. This mechanism may participate in B-cell transformation by HCV. B-cell transformation would not need to require direct infection of B-cells by HCV as this interaction takes place between extracellular E2 expressed on the virion and surface CD81.

As regards to hepatitis C virus, few experimental data support the hypothesis of a direct transformation mechanism accounting for HCV-associated lymphomagenesis. In *in vitro* studies, CD81 has been shown to be an entry receptor for HCV and could be involved in infection of B cells by the virus.^26^ *In vitro*, infection of B-cell lines with HCV leads to somatic mutations of several oncogenes and tumor suppressor genes such as *p53*, *beta-catenin* and *Bcl*6.^27^,^28^ The expression of the HCV core protein (C) and non-structural protein 3 (NS3) has been associated with the induction of nitric oxide synthase (NOS) which might be responsible for these mutations (**Figure 3A**). Similarly, frequent chromosomal polyploidy in peripheral blood mononuclear cells (PBMC) from HCV-positive patients was recently demonstrated, as well as in splenocytes from HCV core protein-expressing transgenic mice, suggesting that HCV infection may inhibit the mitotic checkpoint.^29^ *In vivo*, as it lacks a reverse transcriptase, HCV requires the cellular machinery to efficiently produce negative strand from positive strand viral DNA. Therefore, identification of negative strand RNA sequences in cells is indicative of active virus replication. Using a specific and sensitive method for HCV minus strand RNA detection, Sansonno *et al.* demonstrated active HCV replication in PBMC in nearly half of HCV-positive MC patients whereas no sign of infection was found in HCV-infected individuals without MC.^30^

Notably, in 7 patients with B-NHL without MC, active HCV replication was not found. So far, direct infection of the malignant clone has been described in a single case of B-cell lymphoma associated with HCV.^31^ Therefore, although it can infect B cells *in vivo* and despite an association between PBMC infection and MC, an etiological role of the virus in HCV-associated lymphoproliferative disorders by a direct transformation mechanism is poorly supported by those data as half of MC patients and virtually all NHL patients do not display active HCV replication in PBMC or in the malignant clone respectively.

On the contrary, many studies support the role of HCV as an indirect transformation agent by chronically stimulating B-cell immunologic response and ultimately leading to overt lymphoma in some cases. In this regard, data on the association between MC and NHL have been of great interest. Frequent polyclonal or monoclonal B-cell proliferation can be detected in the blood, bone marrow or liver biopsies of HCV patients and association with type II MC (characterized by a monoclonal IgM with anti-IgG – *i.e.* a rheumatoid factor - activity) has been clearly demonstrated. Hence, 50 to 90% of patients with MC harbor a positive HCV serology and conversely, nearly 40–50% of HCV patients exhibit circulating cryoprecipitating complexes. Along with data on the response of lymphoma to therapy, antiviral treatment has been associated with disappearance of MC and in many cases of B-cell clones.^32^–^35^ About 8 to 10% of patients with cryoglubulinemia ultimately develop a frank lymphoproliferative disorder and recent data demonstrated a 35 times increased risk of lymphoma for cryoglobulinemia carriers compared with general population. Nevertheless, NHL does not always evolve out of MC in what can be thought of as a stepwise evolution and many issues still need to be further elucidated.

Data from V(D)J region analyses among patients with MC or frank NHL also brought new insights into the pathological process from HCV-infection to lymphoma. Restricted usage of V_H_1-69 and V_K_3-20/15 regions have been demonstrated, for instance, among patients with MC and HCV-associated NHL thereby giving strong support to an antigenic selection driven process underlying lymphoma development in HCV-positive patients.^36^,^37^ Sequencing of IgV regions also revealed that they constitute a target for SHM, suggestive of a maturation process under antigenic stimulation.^37^ Furthermore, De Re *et al.* showed BCR from some HCV-related patients recognized both IgG-Fc and HCV-NS3 protein rising the possibility that rheumatoid factor might emerge from cross-reaction between a virus-associated epitope and IgG autoantigen.^38^ Cross-reactivity between E2 directed antibodies and anti-IgG IgM RF in type II MC has also been demonstrated.^39^ One may imagine that Ig-HCV immunocomplexes in chronically infected patients would amplify the phenomenon by stimulating cross-reactive RF producing clones. Other recent data support a major role for HCV envelope glycoprotein E2 in indirect transformation. Not only can it promote HCV cellular entry by binding to CD81 on B-cell surface but when E2 binds to B cells via CD81, this latter then associates with CD19 and CD21, forming a complex that lowers the activation threshold.^40^ The synergy between CD81-CD19-CD21 complex signaling and BCR cross-activation by envelope glycoprotein E2 and/or NS3 is thought to promote B-cell proliferation, emergence of autoreactive clones, genomic instability and NHL development. To further support this hypothesis, direct infection of lymphocytes by HCV would not be necessary *in vivo* to induce somatic mutations of several oncogenes and tumor suppressor genes such as *p53*, *beta-catenin* and *Bcl*6 as recombinant HCV E2 binding to surface CD81 has also been shown to induce somatic hypermutation of the immunoglobulin gene locus (**Figure 3B**).^41^ As the E2 glycoprotein is expressed on the virion surface, this mechanism of mutagenesis would not require direct infection of B cells by HCV.

Altogether, those biological data have led to the concept of a multi-step lymphomagenesis process in HCV-related B-cell clonal disorders by an indirect transformation mechanism.

## Perspective: towards a multi-step lymphomagenesis model

The finding that MC represents an independent risk factor for the development of NHL in HCV-infected patients,^42^ as well as the finding that, among HCV-infected patients with NHL, antiviral treatment was more effective in those with associated MC^16^ lend support to a model in which HCV would induce a protracted stimulation of antigen-specific B-cell clones, leading to MC and in a subset of patients to overt lymphomas.

Besides chronic antigenic stimulation, cytokines and growth factors produced within the inflammatory context of chronic infection are now suspected to play a key role in B-cell transformation. As a matter of fact, BAFF (for B-cell activating factor of the TNF family) has been described as a critical survival factor for B cells, promoting their activation and maturation, mainly through the nonclassical NF-κB pathway.^43^–^47^ BAFF has also been shown to promote the survival of autoreactive B-cell clones in case of abnormal production thus triggering autoimmune diseases.^48^ In HCV infection, BAFF deregulation has been demonstrated, consistent with a role of the virus as a trigger for BAFF upregulation possibly predisposing to B-cell proliferation and clonal expansion.^49^,^50^ A role for BAFF in promoting B-cell survival in an autocrine regulation loop has also been suggested in B-cell lymphoproliferative disorders. Hence, MC HCV-positive patients with NHL displayed higher BAFF levels and more frequently upregulated BAFF levels than MC HCV-positive counterparts without NHL.^51^ Studying other pro-inflammatory cytokines such as IL-17 might also be of interest as this cytokine has recently been described to act in synergy with BAFF in human B-cell survival, proliferation and differentiation into immunoglobulin-secreting cells.^46^ Similarly to BAFF, higher IL-17 serum levels were also demonstrated in patients suffering from systemic lupus erythematosus compared to healthy donors^46^ and a role of IL-17-producing T helper cells in other autoimmune disorders such as rheumatoid arthritis, psoriasis or multiple sclerosis has been suggested.^52^ A recent study demonstrated that PBMC from HCV Ag-positive patients secreted IL-17 in response to stimulation with the HCV nonstructural protein 4 (NS4).^53^ However, NS4 also induced TGF-β and IL-10 expression at high levels by monocytes from HCV-infected patients and those cytokines were shown to significantly suppressed NS4 specific human Th17 cells. The balance between IL17 expected role in protective immunity to HCV and its demonstrated role in inducing autoimmunity has to be further studied in MC and NHL HCV-positive patients to bring new insights into the pathogenesis of these disorders.

Concerning oncogenic events leading to NHL development, cytogenetic information on HCV-associated lymphomas is scarce. Matteucci *et al.* recently studied genomic imbalances in low- and high-grade HCV-related lymphomas and found trisomy 3 or +3q in 4 out of 6 splenic MZL as typically reported in low-grade splenic non HCV-related MZL.^54^ As those 3q+ splenic MZL exhibited an indolent clinical course and regressed after HCV eradication, and since trisomy 3 was also found in circulating B cells of one patient with MC without lymphoma, 3q gain is not supposed to be oncogenic *‘per se’*. Conversely, 2q loss was associated with more aggressive B-cell lymphomas (4 out of 5 DLBCL) with no response to antiviral treatment. Of note, 2 of these DLBCL were derived from low-grade underlying lymphoma. Regarding IgH/BCL2 translocations, unexpected results in HCV-related NHL have been published so far. Hence, whereas the presence of IgH/BCL2 clones is frequent in HCV-infected patients, especially when associated to MC, the translocation was not shown to be any more frequent among HCV-infected NHL patients than among HCV-negative NHL patients.^55^–^57^ This finding suggests that IgH/BCL2, which is present at a low frequency in healthy subjects,^58^ may be amplified by a bystander effect due to a non specific stimulation of the immune system rather than being an additional oncogenic hit that leads to malignant transformation.

Response to treatment from our cohort of patients illustrated the hypothesis of additional oncogenic events driving oligoclonal proliferation in MC to monoclonal expansion in lymphoma. Accordingly, despite the efficacy of antiviral treatment in a majority of patients, leading to complete virological and hematological responses including the disappearance of detectable MC, the B-cell clones were still detectable in all patients.^16^ Those results contrasts with previous findings in HCV-associated patients with MC 32,59 where B-cell clones were not detectable anymore in case of complete virological response. This may reflect differences in oncogenic potential between HCV-driven MC-producing clones and SLVL malignant B cells. In the latter, B-cell clones are therefore likely to have accumulated unknown additional oncogenic events, inducing a survival benefit for this expanded clonal population that remains however still dependent upon antigenic stimulation as demonstrated by response to antiviral treatment.

Aside from SLVL and other low-grade B-cell NHL, HCV is epidemiologically associated with DLBCL.^3^,^4^ We have recently shown that DLBCL in HCV-positive patients were more frequently transformed from low-grade B-cell lymphomas than DLBCL in HCV-negative patients.^60^ The finding that splenic involvement was more frequent in HCV-positive cases also supports a possible transformation from an underlying low-grade B-cell NHL. Furthermore, some differences exist in the clinical outcome of these transformed DLBCL. Surprisingly, patients in the HCV-positive group exhibited a longer event-free survival although the overall survival was significantly shorter in HCV positive patients.^60^ These findings suggest a difference in the pathophysiological mechanisms underlying lymphomagenesis in HCV-associated DLBCL, and, by extension, a causal role for HCV in this lymphoma subtype. Therefore, DLBCL might be seen as the ultimate stage of lymphomagenesis in HCV-positive patients, ranging from indolent lymphoma responsive to therapy like SLVL to aggressive disease independent of antigenic stimulation like DLBCL.

## Conclusion:

Among the many extrahepatic manifestations of HCV, the interactions of the virus with B cells and their subsequent diseases are major consequences of chronic HCV infection. In some cases, the restricted B-cell response to HCV might undergo an oncogenic event giving survival advantage to a subclonal population, which may ultimately lead to a frank lymphoproliferative disorder. Evidence indicates a potential infection of the B-cell compartment in HCV-positive patients but it is likely an indirect transformation process that accounts for HCV-associated lymphoproliferative disorders.

HCV associated MC, low-grade B-cell lymphomas and particularly SLVL and a subset of large B-cell lymphomas can fit in a continuum whereby chronic antigenic stimulation by persistent viral replication leads to progressive and antigen-driven B-cell transformation. These different stages could correspond to a step-by-step model of B-cell lymphomagenesis, ultimately leading to complete transformation and loss of antigen-dependence as seen in DLBCL. MC could thus be viewed as a marker of antigen-dependence of the lymphoproliferation. Several lines of evidence strongly suggest that antiviral therapy should be considered as first-line therapy in HCV-associated lymphomas, especially in the presence of MC. Antiviral therapy could also potentially benefit HCV patients with DLBCL by reducing liver injury inflicted by persistent HCV replication, and possibly by reducing the role of HCV as an oncogenic promoter.

Because of its clinical implications, HCV related low grade lymphoma should be classified as a special entity as it has been proposed in the World Health Organization lymphoma classification for T-cell lymphoproliferation related to HTLV-1.

## Figures and Tables

**Figure 4.**
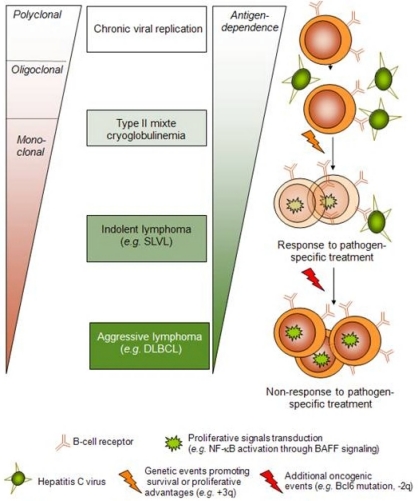
**Hypothetical model of progression from mixed cryoglobulinemia to lymphoma.** Stimulation of auto-reactive, rheumatoid factor producing and HCV-E2 or NS3 cross-reactive specific B cells might lead to a first-step lymphoproliferative development such as in type II mixed cryoglobulinemia (MC). Pro-inflammatory cytokines, such as BAFF (also called B lymphocyte stimulator, BLyS), a potent stimulator of B-cell survival and proliferation, are thought to play an important role in this mechanism. Eradication of the antigenic source at this stage by interferon with or without ribavirin leads to a decrease of the cryoglobulin load and of the clonal population. Additional oncogenic events may occur in the proliferating cryoglobulin-producing population and lead to transformation into NHL such as marginal zone lymphoma with villous lymphocytes (SLVL). Such transformed B cells would still remain dependent upon antigen stimulation as attested by the regression of the tumor after HCV eradication but without full disappearance of the clonal population. Further oncogenic events, such as 2q loss, may lead to transformation into high-grade lymphoma.

**Table 1.** Main therapeutic studies reporting effect of antiviral treatment on HCV-associated NHL

	**Number of patients (n)**	**Age at diagnosis (median or mean)**	**Type II MC (n)**	**Type of lymphoma (n)**	**Treatment (n)**		**Hematological response (n)**			
					IFN	IFN/RBV	CR	PR	SD	PD
Hermine *et al.* 2002^14^	9	55.0	6	SLVL (9)	9	0‡	7	-	2	-
Kelaidi *et al.* 2004^17^	8	50.0	7¶	Splenic MZL (2) SLVL (3) Extranodal MZL (2) Others∑ (2)	1	7	5†	-	2	1
Saadoun *et al.* 2005^16^	18	58.0	18	SLVL (18)	8	10	14	4	-	-
Vallisa *et al.* 2005^18^	13	57.6	5	Nodal MZL (2) Splenic MZL (4) Extranodal MZL (2) Follicular (1) Plasmacytoid (4)	0	13	7	2	2	1∞

‡ Two patients with uncomplete virological response had a subsequent treatment with both IFN and RBV and displayed a concomitant hematological response,

∞ One patient had no available data on response status,

¶ Last patient had type III MC,

∑ Including one leukemic MZL and one disseminated MZL,

† Two patients needed additional PEG IFN + RBV courses to achieve CR

Abbreviations: MC, mixed cryoglobulinemia; IFN, interferon; RBV, ribavirin; CR, complete response; PR, partial response; SD, stable disease; PD, progressive disease; SLVL, splenic marginal zone lymphoma with villous lymphocytes; MZL, marginal zone
